# Supplementary material for: Discovery of the Inhibitor Targeting the SLC7A11/xCT Axis through In Silico and In Vitro Experiments
Source: Int J Mol Sci. 2024 Jul 29;25(15):8284. doi: 10.3390/ijms25158284 (PMC13231825; doi:10.3390/ijms25158284)
Supplement: Supplementary file 1 [file ijms-25-08284-s001.zip › ijms-3112531-Supplementary materials.pdf]

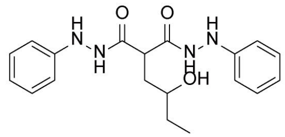

1

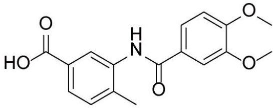

2

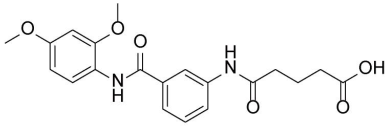

3

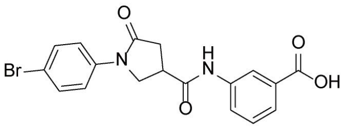

4

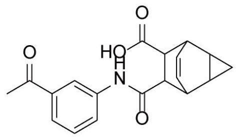

5

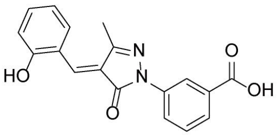

6

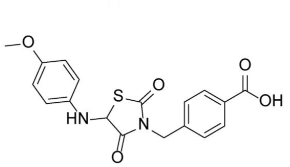

7

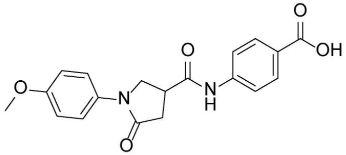

8

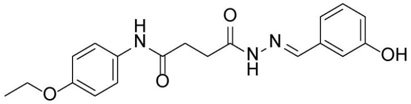

9

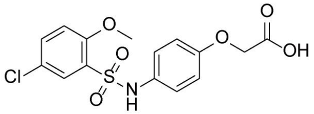

10

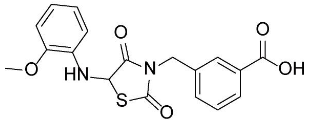

11

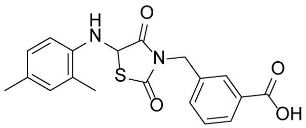

12

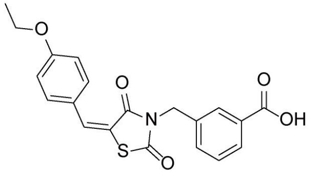

13

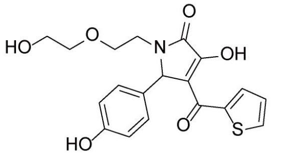

14

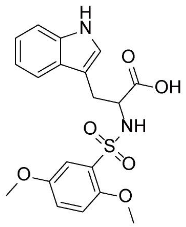

15

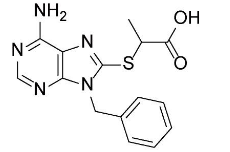

16

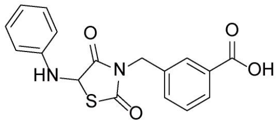

17

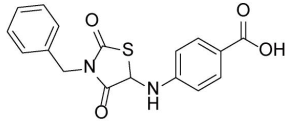

18

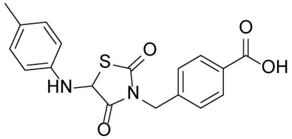

19

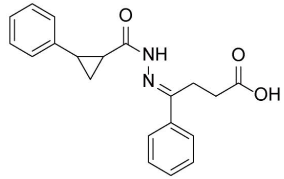

20

Figure S1. The final 20 potential compounds obtained from virtual screening.

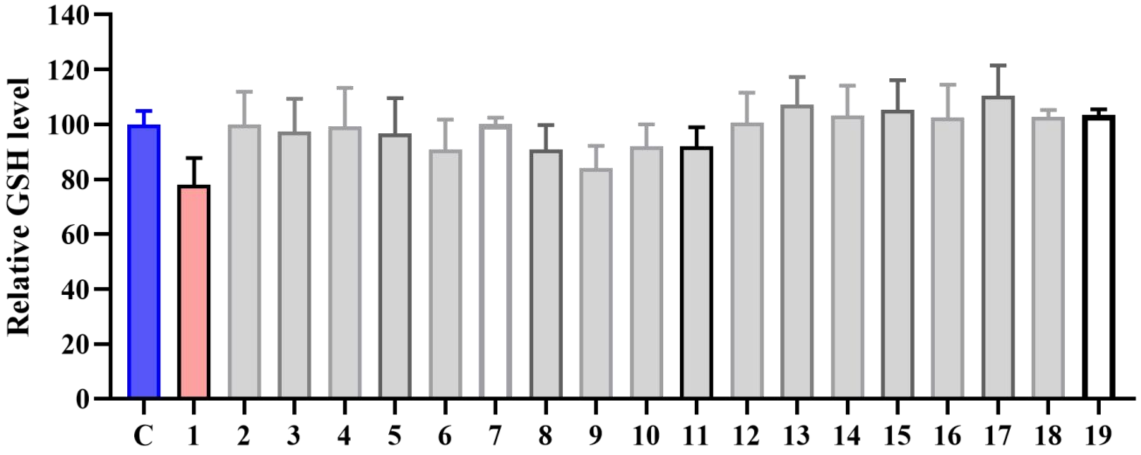

Figure S2. Effect of 19 compounds at 15 μM on intracellular GSH levels in HeLa cells.

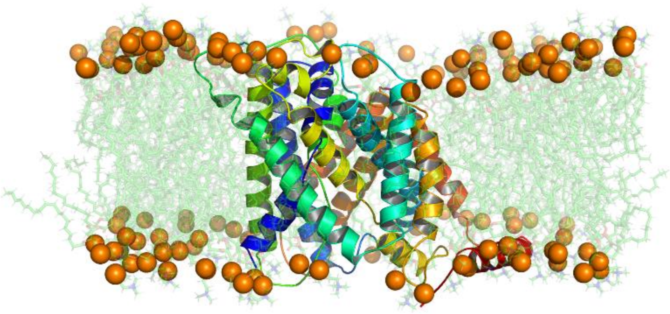

Figure S3. The modeling image of SLC7A11, where orange atoms represent phosphorus (P) atoms, green represents the phospholipid bilayer, and the colored portions are the protein components.

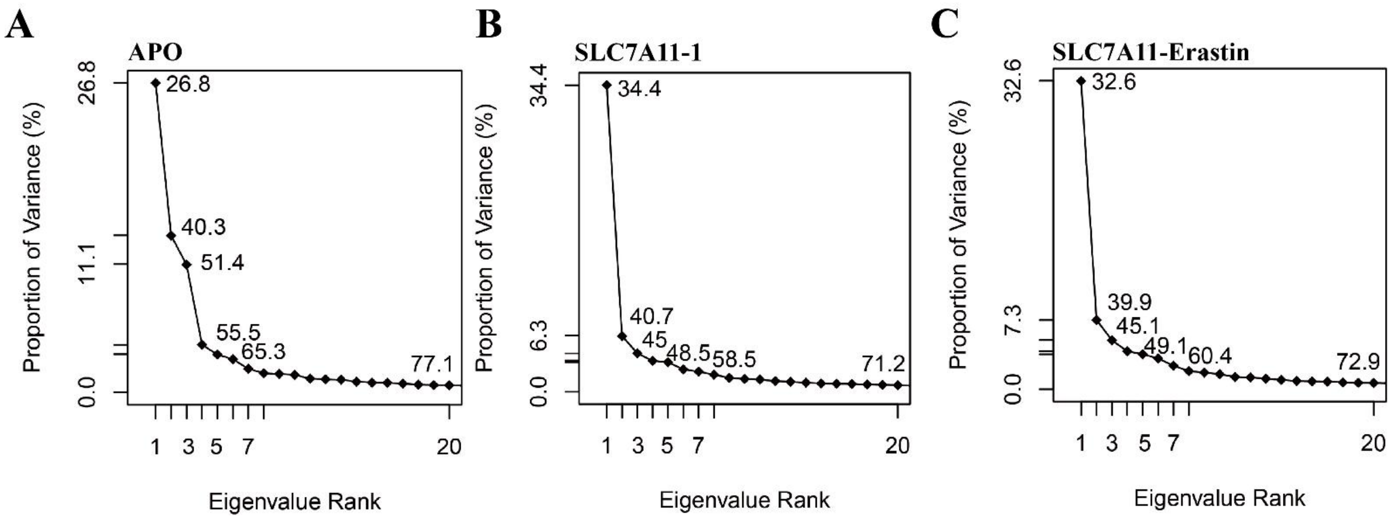

Figure S4. The percentage of variance of eigenvectors graphs.

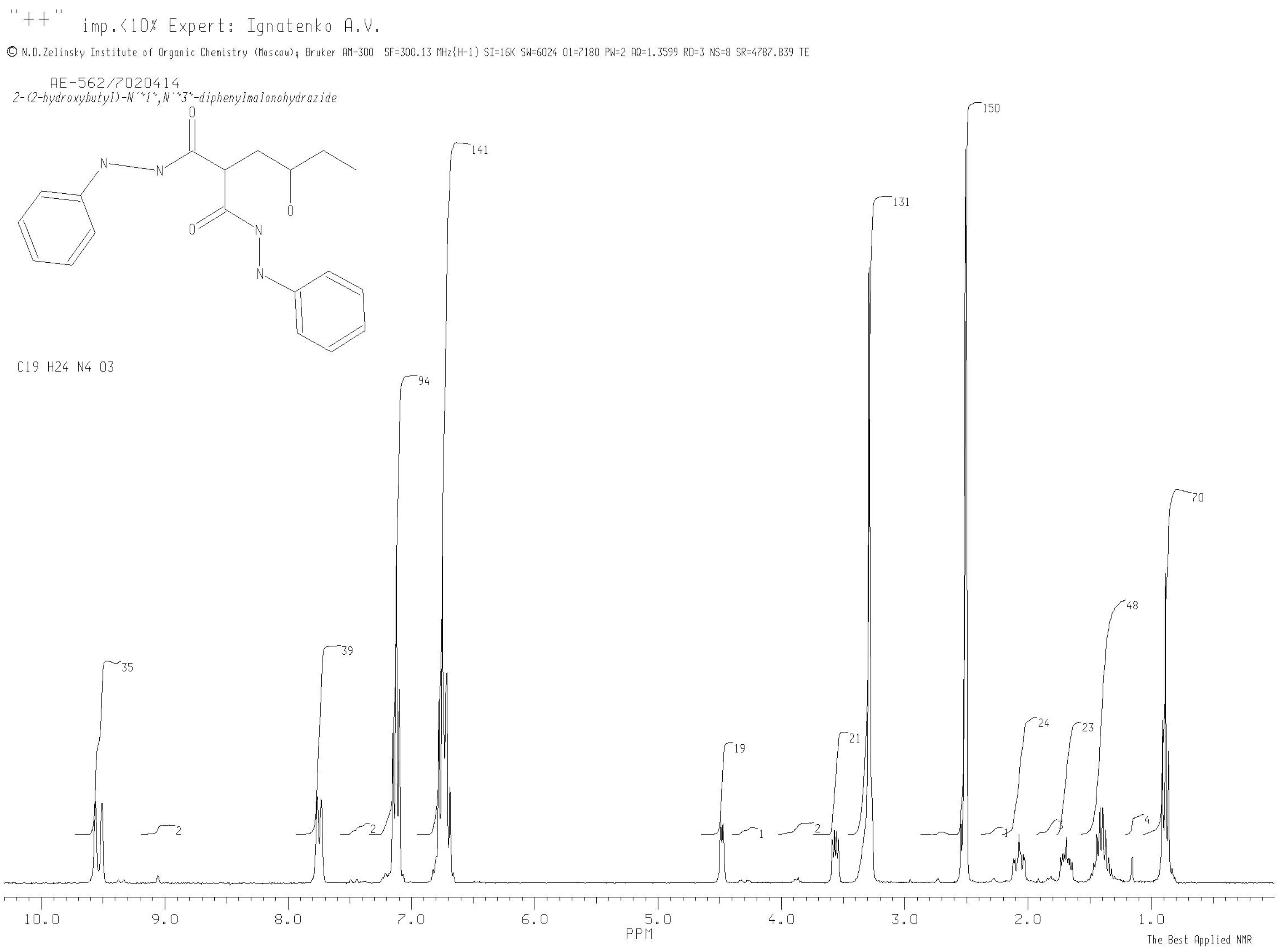

Figure S5. 1H NMR spectra of compound 1.

File:9900140085231 ID:AK-918/40085231 Description:C17H17NO5
Instrument:LC/MS A Vial:1:17 Date:15-Sep-2004

Printed: Mon Sep 20 14:01:41 2004

Sample Report (continued):

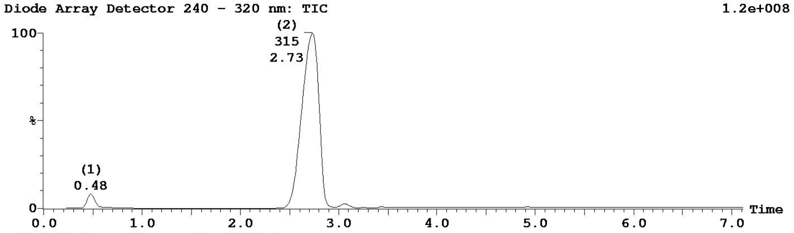

Peak Number	Time	Area %Total
1	0.48	3.48
2	2.73	95.53
3	3.06	0.99

Peak ID	Time	Mass Found	BPM
2	2.73	315.11	316

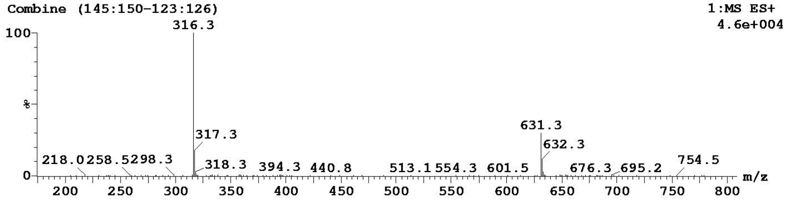

Peak ID	Time	Mass Found	BPM
3	3.06		350

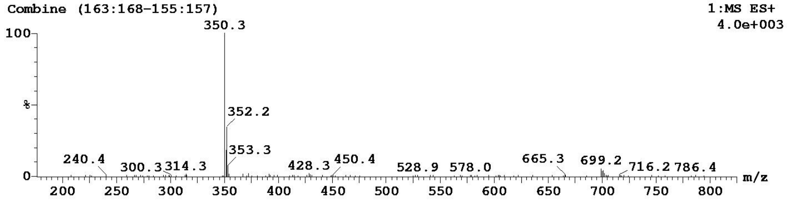

Figure S6. 1H NMR spectra of compound 2.

Sample Report (continued):

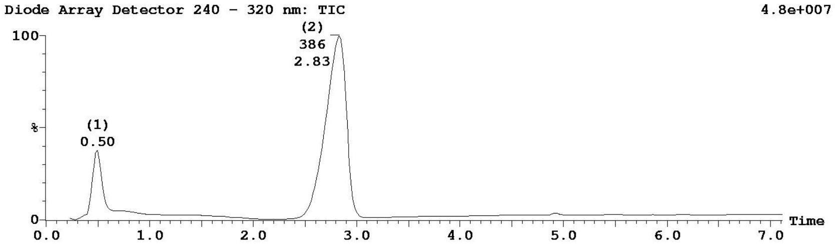

Peak Number	Time	Area %Total
1	0.50	15.74
2	2.83	84.26

Peak ID	Time	Mass Found	BPM
1	0.50		265

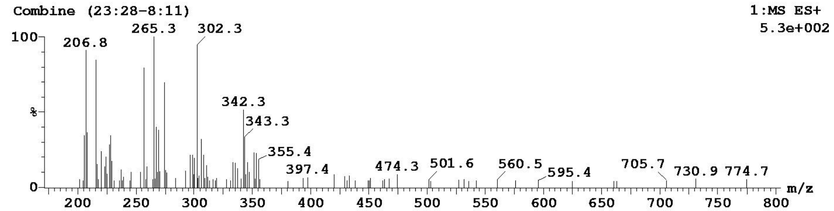

Peak ID	Time	Mass Found	BPM
2	2.83	386.15	387

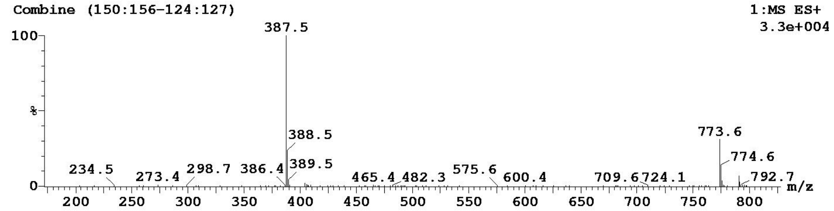

Figure S7. 1H NMR spectra of compound 3.

Specs Page 36
File:9900142815548 ID:AK-918/42815548 Description:C18H15BrN2O4
Instrument:LC/MS B Vial:2:36 Date:16-Sep-2004

Printed: Mon Sep 20 14:16:49 2004

Sample Report (continued):

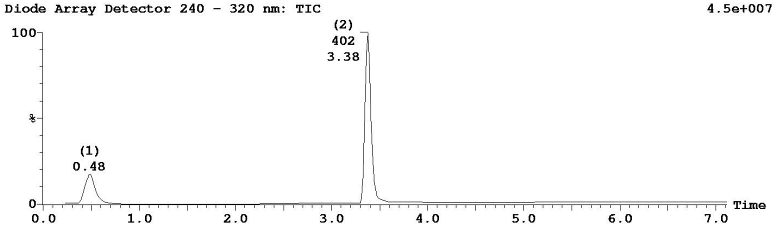

Peak Number	Time	Area %Total
1	0.48	23.24
2	3.38	76.76

Peak ID	Time	Mass Found	BPM
1	0.48		257

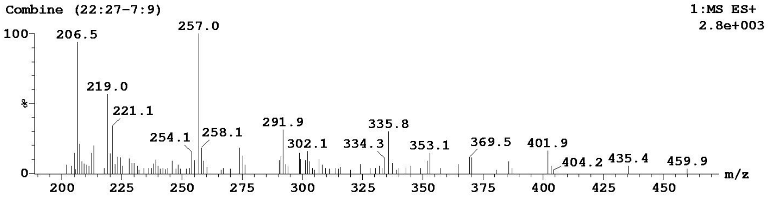

Peak ID	Time	Mass Found	BPM
2	3.38	402.02	405

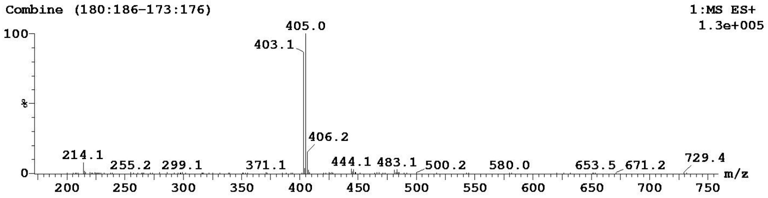

Figure S8. 1H NMR spectra of compound 4.

Sample Report (continued):

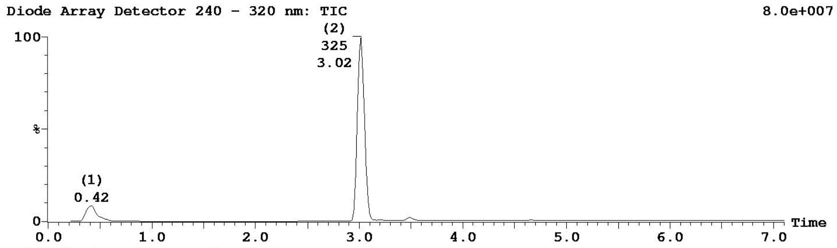

Peak Number	Time	Area %Total
1	0.42	11.55
2	3.02	88.45

Peak ID	Time	Mass Found	BPM
1	0.42		265

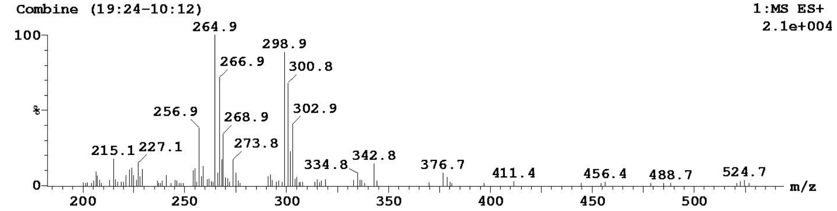

Peak ID	Time	Mass Found	BPM
2	3.02	325.13	326

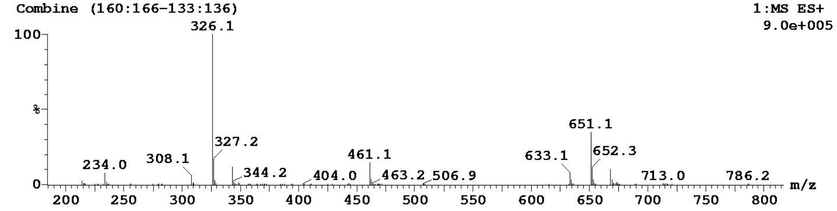

Figure S9. 1H NMR spectra of compound 5.

Sample Report (continued):

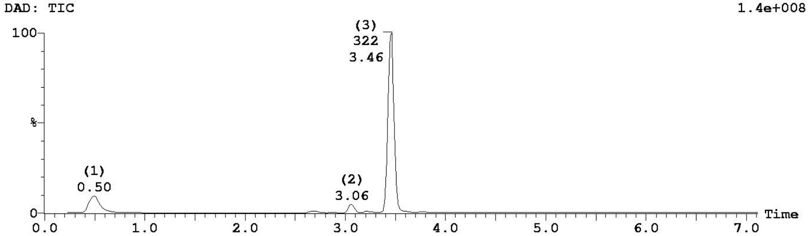

Peak Number	Time	Area %Total
1	0.50	14.20
2	3.06	3.62
3	3.46	82.18

Peak ID	Time	Mass Found	BPM
2	3.06		541.2

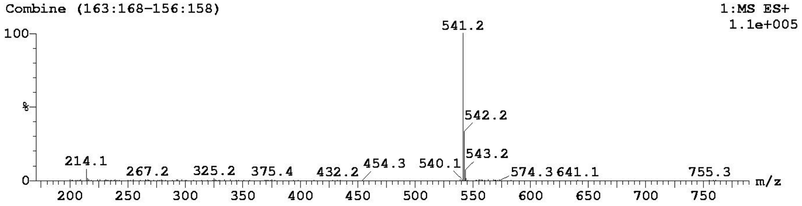

Peak ID	Time	Mass Found	BPM
3	3.46	322.10	323.1

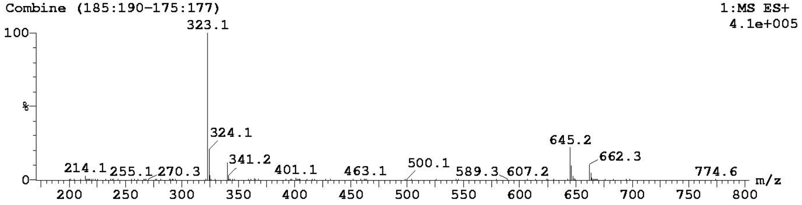

Figure S10. 1H NMR spectra of compound 6.

Specs **info@specs.net, www.specs.net** Page 35

File:9900143208223 ID:AT-057/43208223 Description:C18H16N2O5S

Vial:7:3,C Date:15-Sep-2015 Time:16:39:37

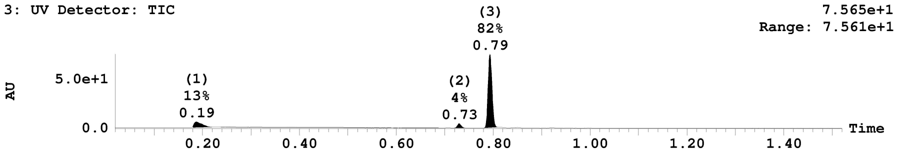

Peak Number	Compound	Time	AreaAbs	Area %Total	Width	Height	Mass Found
1		0.19	1e+005	13.06	0	6e+006	
2		0.73	4e+004	4.49	0	4e+006	
3	Found	0.79	7e+005	82.45	0	7e+007	372.08

Peak ID	Time	Mass Found	BPM
1	0.19		45.84

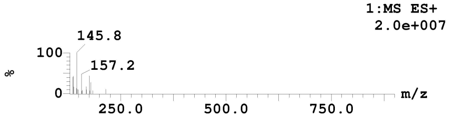

Peak ID	Time	Mass Found	BPM
2	0.73		82.56

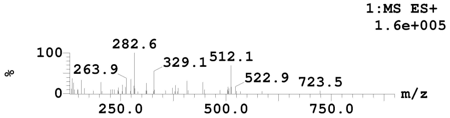

Peak ID	Time	Mass Found	BPM
3	0.79	373.08	72.95

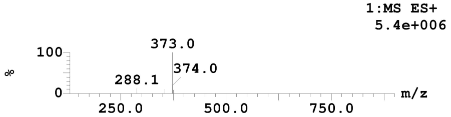

Peak ID	Time	Mass Found	BPM
3	0.79		11.05

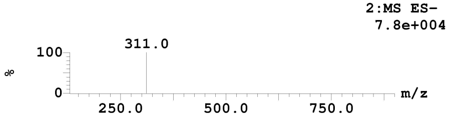

Figure S11. 1H NMR spectra of compound 7.

File:9900242815282	ID:AN-153/42815282	Description:C19H18N2O5
Vial:4:7,H	Date:29-Apr-2008	Time:15:32:22

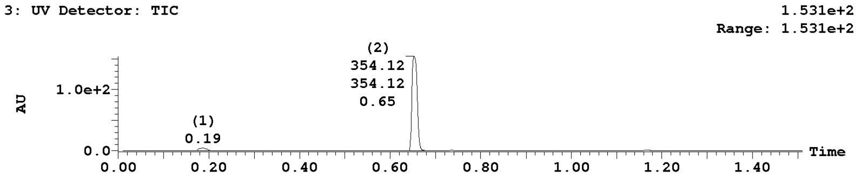

Peak Number	Time	Area %Total
1	0.19	4.37
2	0.65	95.63

Peak ID	Time	Mass Found	BPM
1	0.19		157.01

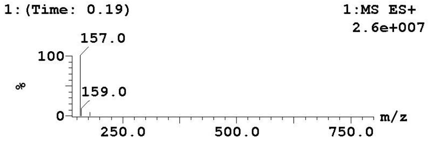

Peak ID	Time	Mass Found	BPM
1	0.19		191.05

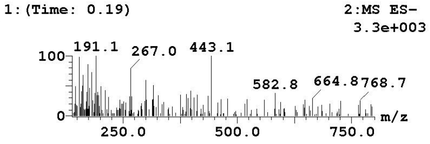

Peak ID	Time	Mass Found	BPM
2	0.65	354.12	355.15

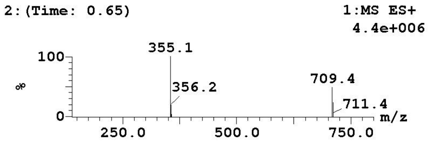

Peak ID	Time	Mass Found	BPM
2	0.65	354.12	353.13

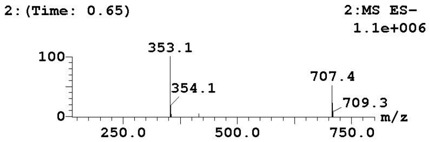

Figure S12. 1H NMR spectra of compound 8.

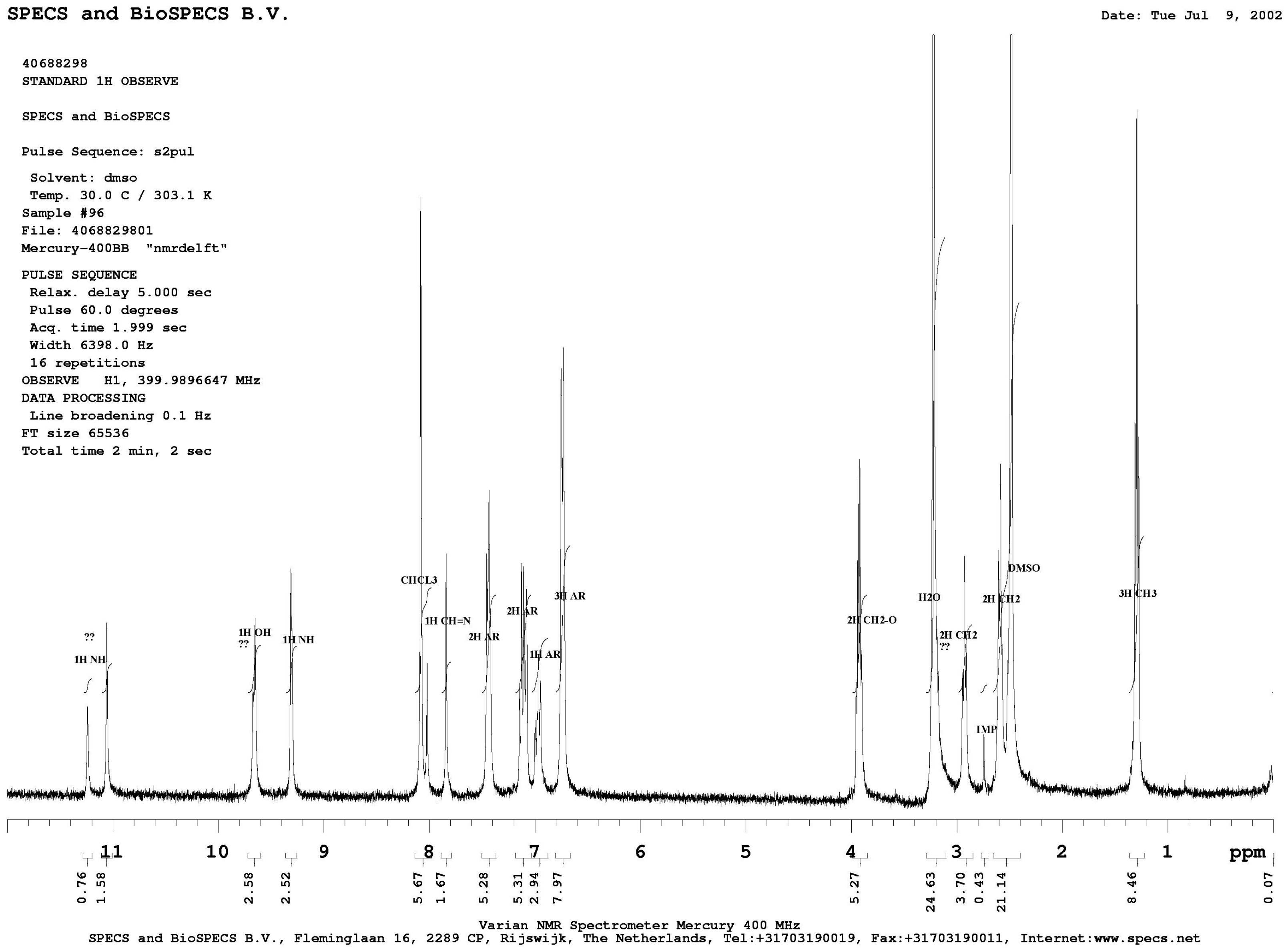

Figure S13. 1H NMR spectra of compound 9.

Specs info@specs.net, www.specs.net Page 227

File:9900143503305 ID:AP-263/43503305 Description:C15H14ClNO6S
Vial:8:15,C Date:26-Jul-2016 Time:23:09:13

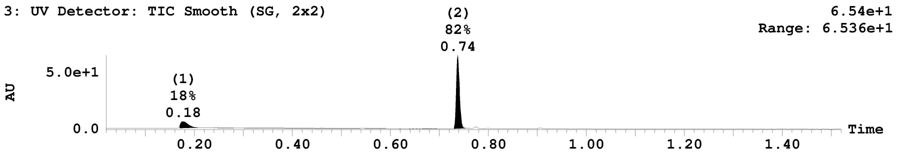

Peak Number	Compound	Time	AreaAbs	Area %Total	Width	Height	Mass Found
1		0.18	1e+005	17.66	0	6e+006	
2	Found	0.74	5e+005	82.34	0	6e+007	371.02

Peak ID	Time	Mass Found	BPM
1	0.18		78.85

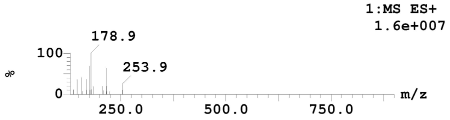

Peak ID	Time	Mass Found	BPM
2	0.74		66.87

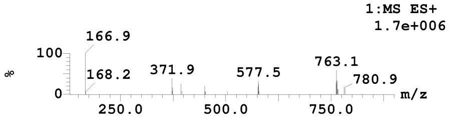

Peak ID	Time	Mass Found	BPM
2	0.74	370.02	70.05

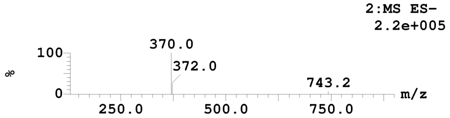

Figure S14. 1H NMR spectra of compound 10.

SPECS and BioSPECS B.V. Date: Mon Oct 29, 2001

40872489
STANDARD 1H OBSERVE

SPECS and BioSPECS

Pulse Sequence: s2pul

Solvent: dmso
Temp. 30.0 C / 303.1 K
Sample #73
File: 4087248901
Mercury-400BB "nmrdelft"

PULSE SEQUENCE
Relax. delay 5.000 sec
Pulse 60.0 degrees
Acq. time 1.999 sec
Width 6398.0 Hz
16 repetitions
OBSERVE H1, 399.9896647 MHz
DATA PROCESSING
Line broadening 0.1 Hz
FT size 65536
Total time 2 min, 2 sec

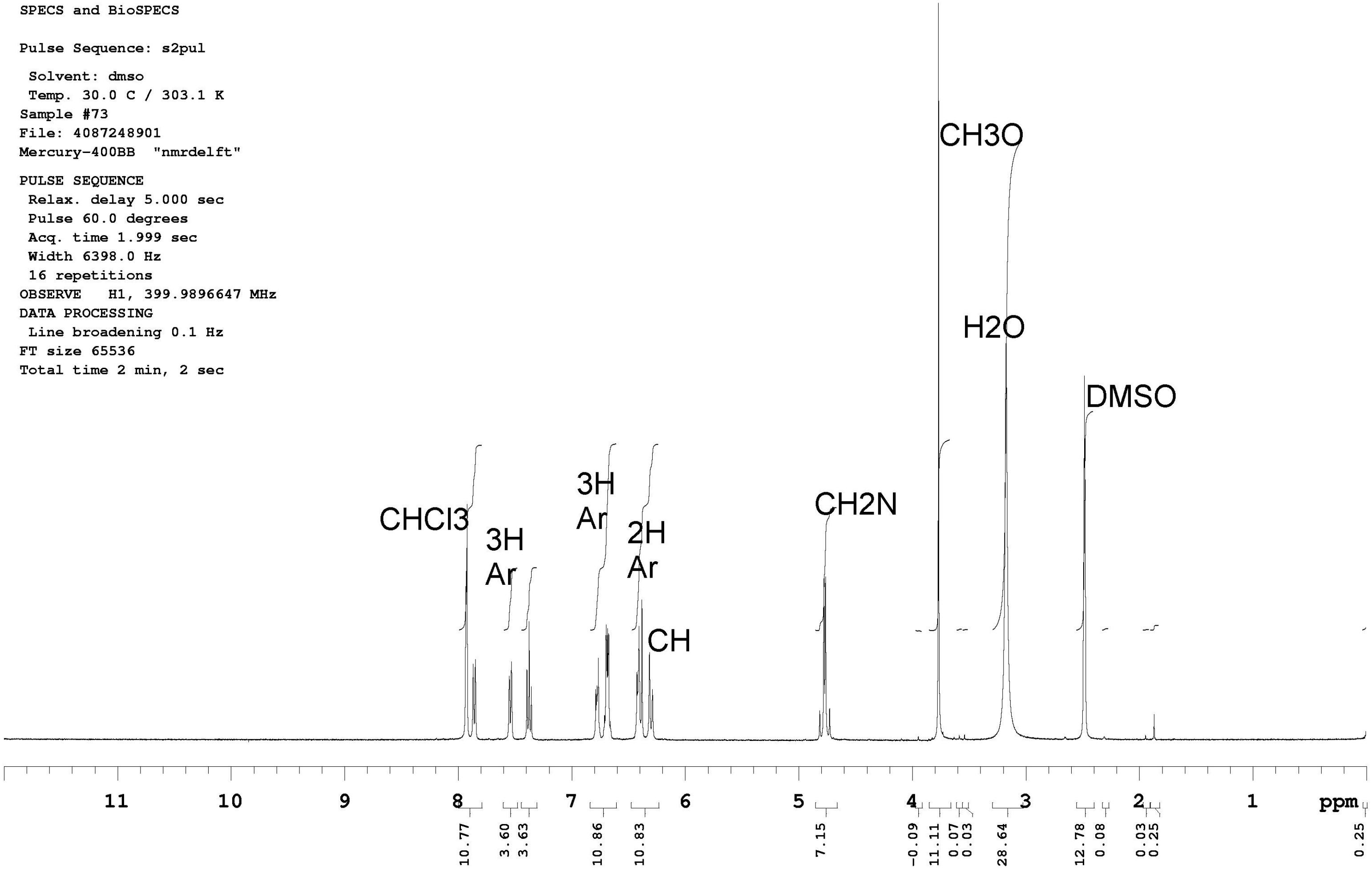

Varian NMR Spectrometer Mercury 400 MHz
SPECS and BioSPECS B.V., Fleminglaan 16, 2289 CP, Rijswijk, The Netherlands, Tel:+31703190019, Fax:+31703190011, Internet:www.specs.net

Figure S15. 1H NMR spectra of compound 11.

SPECS and BioSPECS B.V., The Netherlands Page 15

File:9900140872491	ID:AP-893/40872491	Description:C19H18N2O4S
Vial:1:15	Date:19-Jul-2001	Time:17:36:37

Printed: Mon Jul 30 16:50:49 2001

Sample Report (continued):

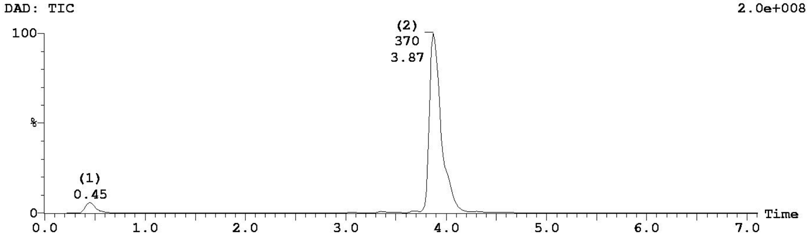

Peak Number	Time	Area %Total
1	0.45	5.63
2	3.87	94.37

Peak ID	Time	Mass Found	BPM
1	0.45		274.0

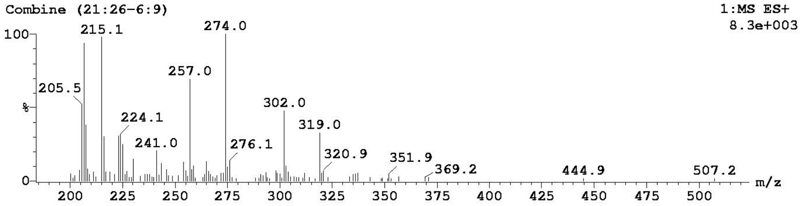

Peak ID	Time	Mass Found	BPM
2	3.87	370.10	371.1

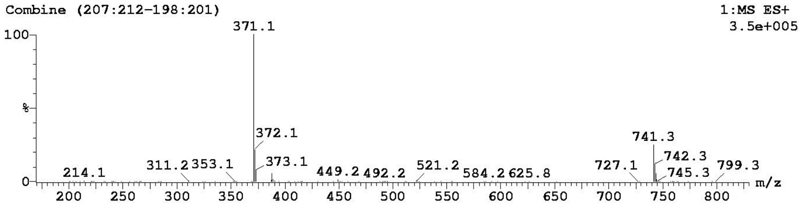

Figure S16. 1H NMR spectra of compound 12.

SPECS and BioSPECS B.V., The Netherlands Page 89

File:9900140872504 ID:AP-893/40872504 Description:C20H17NO5S

Vial:1:89 Date:20-Jul-2001 Time:04:10:11

Printed: Fri Jul 20 16:15:04 2001

Sample Report (continued):

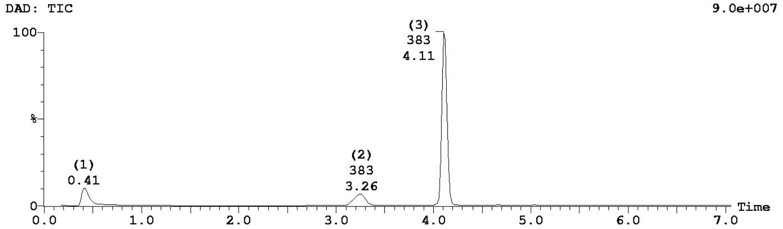

Peak Number	Time	Area %Total
1	0.41	8.91
2	3.26	11.16
3	4.11	79.93

Peak ID	Time	Mass Found	BPM
1	0.41		215.0

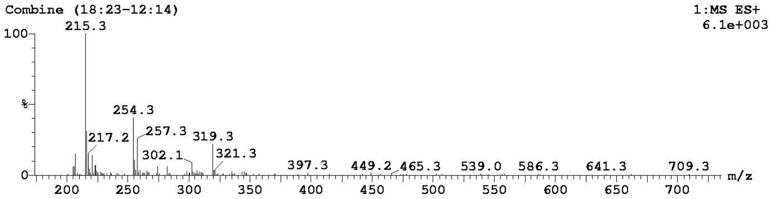

Peak ID	Time	Mass Found	BPM
3	4.11	383.08	401.0

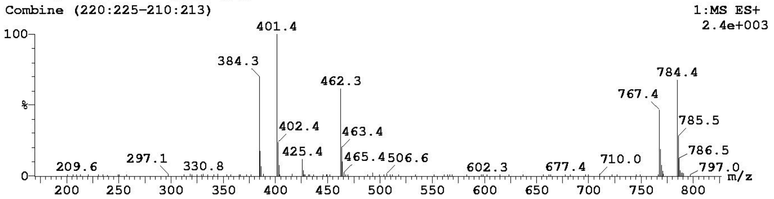

Figure S17. 1H NMR spectra of compound 13.

Specs **info@specs.net, www.specs.net** Page 45

File:9900143372341 ID:AQ-149/43372341 Description:C19H19NO6S

Vial:1:45 Date:30-Nov-2007 Time:22:31:16

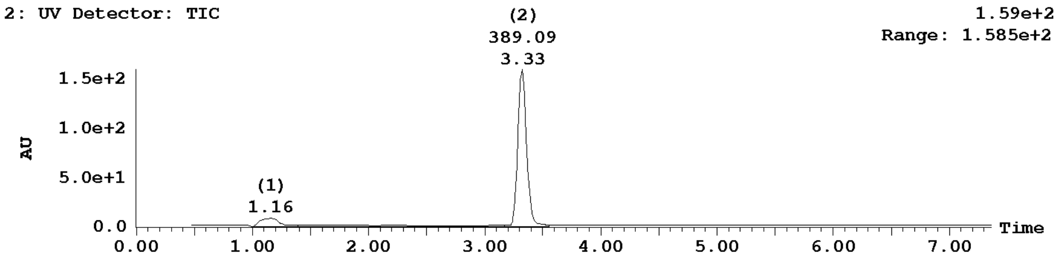

Peak Number	Time	Area %Total
1	1.16	9.11
2	3.33	90.89

Peak ID	Time	Mass Found	BPM
1	1.16		290.95

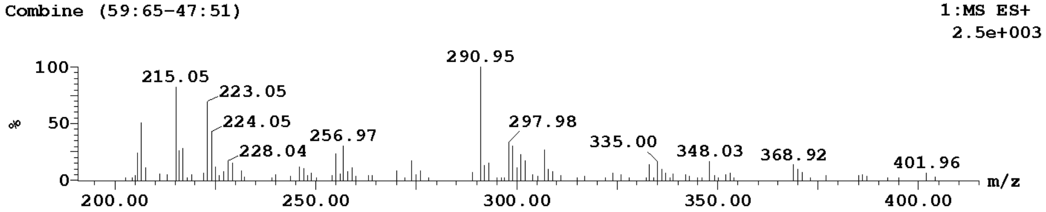

Peak ID	Time	Mass Found	BPM
2	3.33	389.09	390.18

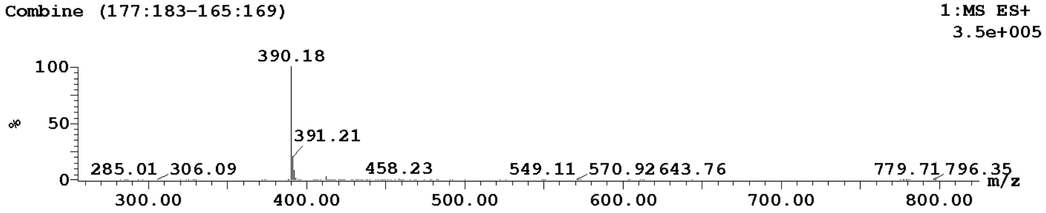

Figure S18. 1H NMR spectra of compound 14.

Specs **info@specs.net, www.specs.net** Page 5

File:9900143490660 ID:AQ-390/43490660 Description:C19H20N2O6S

Vial:8:1,E Date:18-Aug-2015 Time:15:12:04

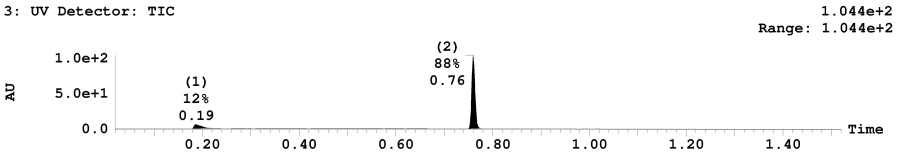

Peak Number	Compound	Time	AreaAbs	Area %Total	Width	Height	Mass Found
1		0.19	1e+005	12.18	0	6e+006	
2	Found	0.76	9e+005	87.82	0	1e+008	404.10, 404.10

Peak ID	Time	Mass Found	BPM
1	0.19		45.85

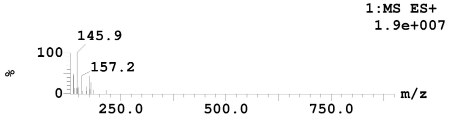

Peak ID	Time	Mass Found	BPM
2	0.76	405.10	04.97

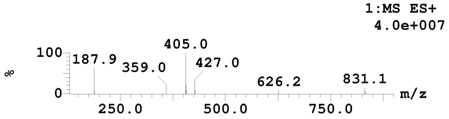

Peak ID	Time	Mass Found	BPM
2	0.76	403.10	03.07

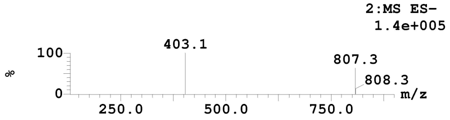

Figure S19. 1H NMR spectra of compound 15.

Specs **info@specs.net, www.specs.net** Page 48
File:9900141598846 ID:AR-422/41598846 Description:C15H15N5O2S
Vial:1:48 Date:05-Oct-2006 Time:18:40:58

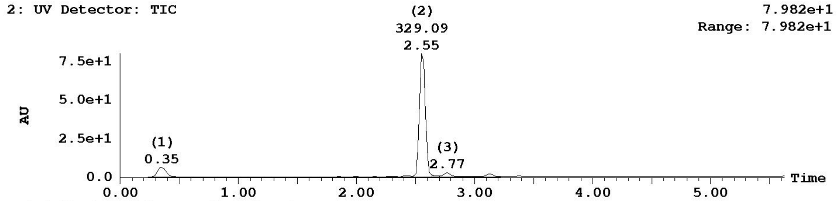

Peak Number	Time	Area %Total
1	0.35	9.37
2	2.55	86.49
3	2.77	2.33
4	3.14	1.81

Peak ID	Time	Mass Found	BPM
2	2.55	329.09	330.26

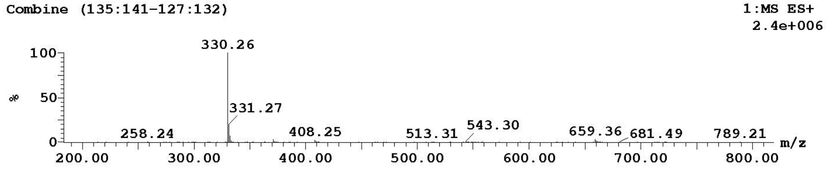

Peak ID	Time	Mass Found	BPM
3	2.77		258.27

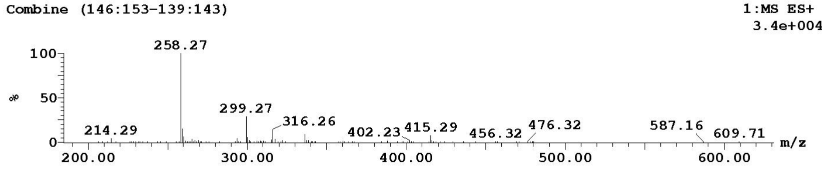

Peak ID	Time	Mass Found	BPM
4	3.14		445.26

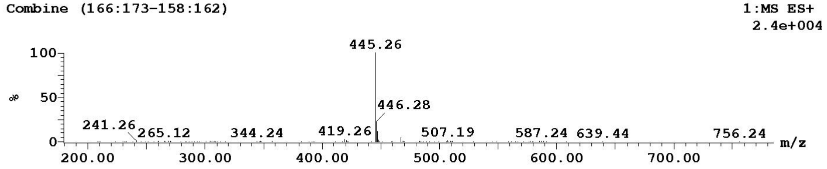

Figure S20. 1H NMR spectra of compound 16.

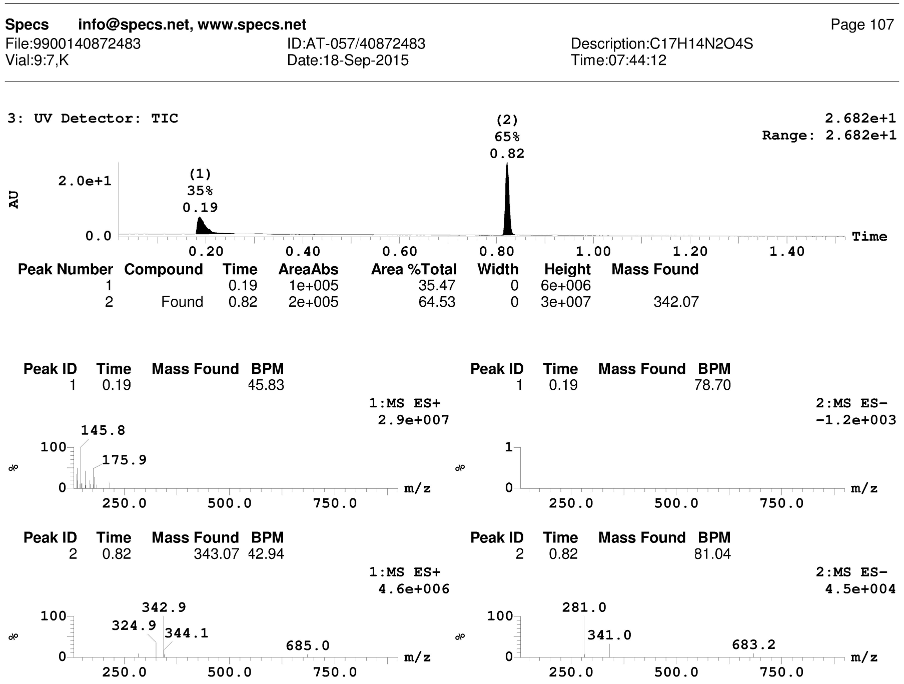

Peak Number	Compound	Time	AreaAbs	Area %Total	Width	Height	Mass Found
1		0.19	1e+005	35.47	0	6e+006	
2	Found	0.82	2e+005	64.53	0	3e+007	342.07

Peak ID	Time	Mass Found	BPM
1	0.19		45.83

Peak ID	Time	Mass Found	BPM
1	0.19		78.70

Peak ID	Time	Mass Found	BPM
2	0.82	343.07	42.94

Peak ID	Time	Mass Found	BPM
2	0.82		81.04

Figure S21. 1H NMR spectra of compound 17.

File:9900142324120 ID:AT-057/42324120 Description:C17H14N2O4S
Vial:8:19,P Date:16-Sep-2015 Time:22:06:17

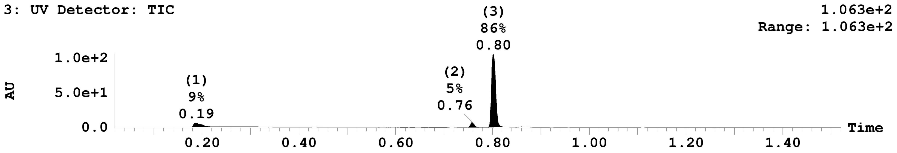

Peak Number	Compound	Time	AreaAbs	Area %Total	Width	Height	Mass Found
1		0.19	1e+005	8.99	0	6e+006	
2		0.76	6e+004	5.05	0	7e+006	
3	Found	0.80	1e+006	85.96	0	1e+008	342.07

Peak ID	Time	Mass Found	BPM
1	0.19		45.86

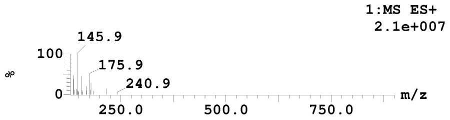

Peak ID	Time	Mass Found	BPM
2	0.76		59.41

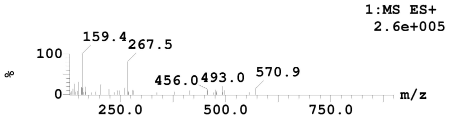

Peak ID	Time	Mass Found	BPM
2	0.76		97.50

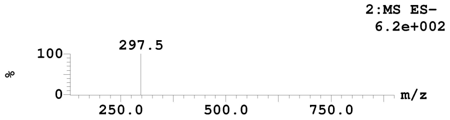

Peak ID	Time	Mass Found	BPM
3	0.80	343.07	42.93

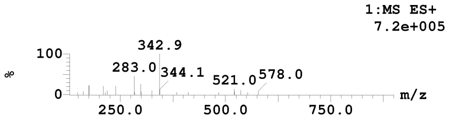

Peak ID	Time	Mass Found	BPM
3	0.80		81.14

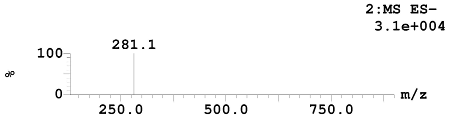

Figure S22. 1H NMR spectra of compound 18.

Specs info@specs.net, www.specs.net Page 28

File:9900143208212 ID:AT-057/43208212 Description:C18H16N2O4S

Vial:7:2,L Date:15-Sep-2015 Time:16:24:41

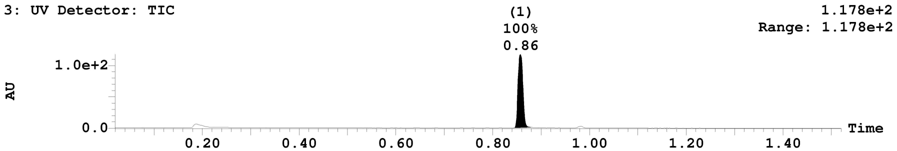

Peak Number	Compound	Time	AreaAbs	Area %Total	Width	Height	Mass Found
1	Found	0.86	1e+006	100.00	0	1e+008	356.08

Peak ID	Time	Mass Found	BPM
1	0.86	357.08	56.97

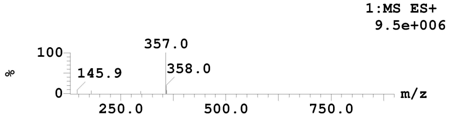

Peak ID	Time	Mass Found	BPM
1	0.86		95.08

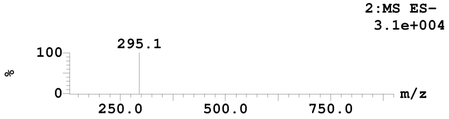

Figure S23. 1H NMR spectra of compound 19.

Table S1. DBI and psF values corresponding to different cluster counts of the SLC7A11-erastin system.

Number of clusters	DBI	pSF
2	1.498305	217.623624
3	1.968536	135.175979
4	2.429449	101.968981
5	2.491442	83.457974
6	2.458398	77.242444
7	2.252899	64.564769
8	2.064265	55.658026
9	2.155889	52.153633
10	2.130915	46.833220

Table S2. DBI and psF values corresponding to different cluster counts of the SLC7A11-c1 system.

Number of clusters	DBI	pSF
2	1.721437	1651.407548
3	1.961998	1082.633158
4	2.040604	1072.055802
5	1.852263	931.063652
6	1.891707	833.841696
7	1.851354	769.127048
8	1.992519	702.399516
9	2.004552	635.755483

10	2.073410	573.791920
